# 3-(2-Fluoro­phenyl­sulfin­yl)-2,4,5,6-tetra­methyl-1-benzofuran

**DOI:** 10.1107/S1600536812035714

**Published:** 2012-08-23

**Authors:** Hong Dae Choi, Pil Ja Seo, Uk Lee

**Affiliations:** a Department of Chemistry, Dongeui University, San 24 Kaya-dong, Busanjin-gu, Busan 614-714, Republic of Korea; b Department of Chemistry, Pukyong National University, 599-1 Daeyeon 3-dong, Nam-gu, Busan 608-737, Republic of Korea

## Abstract

In the title compound, C_18_H_17_FO_2_S, the 2-fluoro­phenyl ring makes a dihedral angle of 85.45 (4)° with the mean plane [r.m.s. deviation = 0.017 (1) Å] of the benzofuran fragment. In the crystal, mol­ecules are linked by weak C—H⋯O and C—H⋯π inter­actions.

## Related literature

For background information and the crystal structures of related compounds, see: Seo *et al.* (2011*a* ▶,*b* ▶).
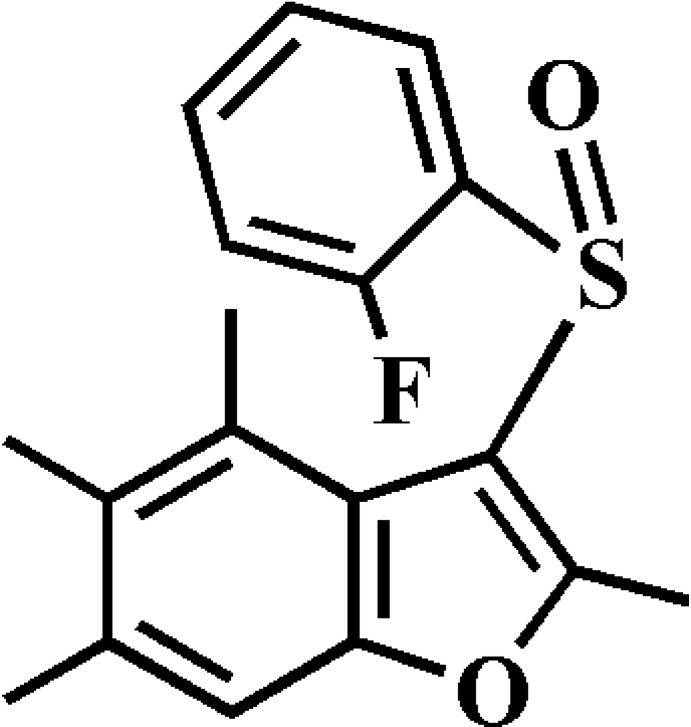


## Experimental

### 

#### Crystal data

C_18_H_17_FO_2_S
*M* *_r_* = 316.38
Monoclinic, 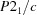

*a* = 14.6851 (4) Å
*b* = 6.0786 (2) Å
*c* = 17.1647 (4) Å
β = 102.223 (1)°
*V* = 1497.47 (7) Å^3^
*Z* = 4
Mo *K*α radiation
μ = 0.23 mm^−1^
*T* = 173 K
0.33 × 0.30 × 0.07 mm

#### Data collection

Bruker SMART APEXII CCD diffractometer
Absorption correction: multi-scan (*SADABS*; Bruker, 2009 ▶) *T*_min_ = 0.593, *T*_max_ = 0.746
24418 measured reflections
3264 independent reflections
2803 reflections with *I* > 2σ(*I*)
*R*_int_ = 0.043

#### Refinement

*R*[*F* ^2^ > 2σ(*F* ^2^)] = 0.038
*wR*(*F* ^2^) = 0.101
*S* = 1.08
3264 reflections
203 parameters
H-atom parameters constrained
Δρ_max_ = 0.28 e Å^−3^
Δρ_min_ = −0.40 e Å^−3^

### 

Data collection: *APEX2* (Bruker, 2009 ▶); cell refinement: *SAINT* (Bruker, 2009 ▶); data reduction: *SAINT*; program(s) used to solve structure: *SHELXS97* (Sheldrick, 2008 ▶); program(s) used to refine structure: *SHELXL97* (Sheldrick, 2008 ▶); molecular graphics: *ORTEP-3* (Farrugia, 1997 ▶) and *DIAMOND* (Brandenburg, 1998 ▶); software used to prepare material for publication: *SHELXL97*.

## Supplementary Material

Crystal structure: contains datablock(s) global, I. DOI: 10.1107/S1600536812035714/lr2079sup1.cif

Structure factors: contains datablock(s) I. DOI: 10.1107/S1600536812035714/lr2079Isup2.hkl

Supplementary material file. DOI: 10.1107/S1600536812035714/lr2079Isup3.cml

Additional supplementary materials:  crystallographic information; 3D view; checkCIF report

## Figures and Tables

**Table 1:** Hydrogen-bond geometry (Å, °) *Cg*1 and *Cg*2 are the centroids of the C2–C7 benzene ring and the C13–C18 2-fluoro­phenyl ring, respectively.

*D*—H⋯*A*	*D*—H	H⋯*A*	*D*⋯*A*	*D*—H⋯*A*
C6—H6⋯O2^i^	0.95	2.40	3.3279 (19)	165
C12—H12*C*⋯*Cg*1^ii^	0.98	2.69	3.486 (2)	138
C16—H16⋯*Cg*2^iii^	0.95	2.70	3.553 (2)	149

Symmetry codes: (i) 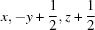; (ii) 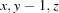; (iii) 
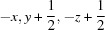
.

**Fig. 1.**
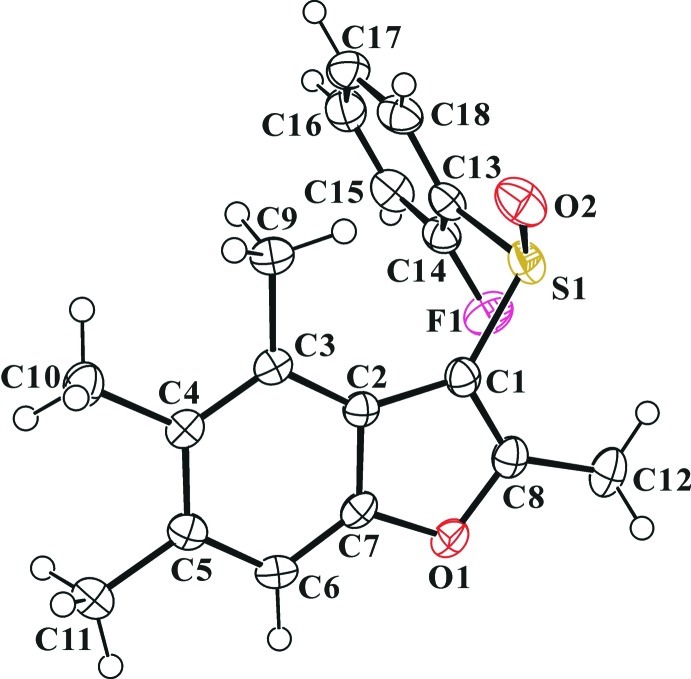
The molecular structure of the title compound. Displacement ellipsoids are drawn at the 50% probability level.

**Fig. 2.**
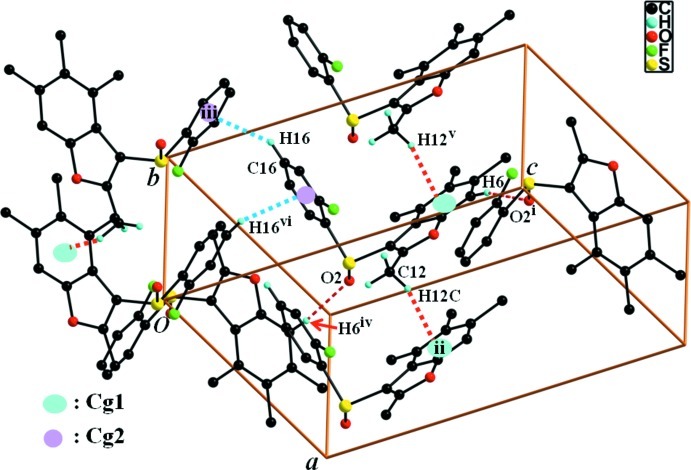
A view of the C—H···O and C—H···π interactions (dotted lines) in the crystal structure of the title compound. H atoms non-participating in hydrogen-bonding were omitted for clarity. [Symmetry codes: (i) x, - y +1/2, z +1/2 (ii) x, y - 1, z (iii) - x, y + 1/2, - z + 1/2 (iv) x, - y + 1/2, z - 1/2 (v) x, y + 1, z (vi) - x, y - 1/2, - z + 1/2.]

## supplementary crystallographic information

### Comment

As a part of our ongoing study of 2,4,5,6-tetramethyl-1-benzofuran derivatives containing 3-phenylsulfinyl (Seo *et al.*, 2011*a*) and 3-(3-fluorophenylsulfinyl) (Seo *et al.*, 2011*b*) substituents, we report herein the crystal structure of the title compound.

In the title molecule (Fig. 1), the benzofuran unit is essentially planar, with a mean deviation of 0.017 (1) Å from the least-squares plane defined by the nine constituent atoms. The dihedral angle between the 2-fluorophenyl ring and the mean plane of the benzofuran ring is 85.45 (4)°. In the crystal structure (Fig. 2), molecules are connected by weak C–H···O and C—H···π interactions (Table 1, Cg1 and Cg2 are the centroids -of the C2–C7 benzene ring and the C13–C18 2-fluorophenyl ring, respectively).

### Experimental

3-Chloroperoxybenzoic acid (77%, 269 mg, 1.2 mmol) was added in small portions to a stirred solution of 3-(2-fluorophenylsulfanyl)-2,4,5,6-tetramethyl-1-benzofuran (330 mg, 1.1 mmol) in dichloromethane (40 mL) at 273 K. After being stirred at room temperature for 3h, the mixture was washed with saturated sodium bicarbonate solution and the organic layer was separated, dried over magnesium sulfate, filtered and concentrated at reduced pressure. The residue was purified by column chromatography (hexane–ethyl acetate, 4:1 v/v) to afford the title compound as a colorless solid [yield 63%, m.p. 464–465 K; *R*_f_ = 0.63 (hexane–ethyl acetate, 4:1 v/v)]. Single crystals suitable for X-ray diffraction were prepared by slow evaporation of a solution of the title compound in ethyl acetate at room temperature.

### Refinement

All H atoms were positioned geometrically and refined using a riding model, with C—H = 0.95 Å for aryl and 0.98 Å for methyl H atoms. *U*_iso_(H) = 1.2*U*_eq_(C) for aryl and 1.5*U*_eq_(C) for methyl H atoms. The positions of methyl hydrogens were optimized rotationally.

### Crystal data

**Table:**

C_18_H_17_FO_2_S	*F*(000) = 664
*M**_r_* = 316.38	*D*_x_ = 1.403 Mg m^−^^3^
Monoclinic, *P*2_1_/*c*	Mo *K*α radiation, λ = 0.71073 Å
Hall symbol: -P 2ybc	Cell parameters from 7371 reflections
*a* = 14.6851 (4) Å	θ = 2.4–28.3°
*b* = 6.0786 (2) Å	µ = 0.23 mm^−^^1^
*c* = 17.1647 (4) Å	*T* = 173 K
β = 102.223 (1)°	Block, colourless
*V* = 1497.47 (7) Å^3^	0.33 × 0.30 × 0.07 mm
*Z* = 4	

### Data collection

**Table:**

Bruker SMART APEXII CCD diffractometer	3264 independent reflections
Radiation source: rotating anode	2803 reflections with *I* > 2σ(*I*)
Graphite multilayer monochromator	*R*_int_ = 0.043
Detector resolution: 10.0 pixels mm^-1^	θ_max_ = 27.0°, θ_min_ = 1.4°
φ and ω scans	*h* = −18→18
Absorption correction: multi-scan (*SADABS*; Bruker, 2009)	*k* = −7→7
*T*_min_ = 0.593, *T*_max_ = 0.746	*l* = −21→21
24418 measured reflections	

### Refinement

**Table:**

Refinement on *F*^2^	Primary atom site location: structure-invariant direct methods
Least-squares matrix: full	Secondary atom site location: difference Fourier map
*R*[*F*^2^ > 2σ(*F*^2^)] = 0.038	Hydrogen site location: difference Fourier map
*wR*(*F*^2^) = 0.101	H-atom parameters constrained
*S* = 1.08	*w* = 1/[σ^2^(*F*_o_^2^) + (0.0453*P*)^2^ + 0.658*P*] where *P* = (*F*_o_^2^ + 2*F*_c_^2^)/3
3264 reflections	(Δ/σ)_max_ < 0.001
203 parameters	Δρ_max_ = 0.28 e Å^−^^3^
0 restraints	Δρ_min_ = −0.40 e Å^−^^3^

### Special details

**Table:**

Geometry. All esds (except the esd in the dihedral angle between two l.s. planes) are estimated using the full covariance matrix. The cell esds are taken into account individually in the estimation of esds in distances, angles and torsion angles; correlations between esds in cell parameters are only used when they are defined by crystal symmetry. An approximate (isotropic) treatment of cell esds is used for estimating esds involving l.s. planes.
Refinement. Refinement of F^2^ against ALL reflections. The weighted R-factor wR and goodness of fit S are based on F^2^, conventional R-factors R are based on F, with F set to zero for negative F^2^. The threshold expression of F^2^ > 2sigma(F^2^) is used only for calculating R-factors(gt) etc. and is not relevant to the choice of reflections for refinement. R-factors based on F^2^ are statistically about twice as large as those based on F, and R- factors based on ALL data will be even larger.

### Fractional atomic coordinates and isotropic or equivalent isotropic displacement parameters (Å^2^)

**Table:**

	*x*	*y*	*z*	*U*_iso_*/*U*_eq_	
S1	0.21698 (3)	0.16904 (7)	0.41644 (2)	0.03042 (13)	
F1	0.03896 (7)	0.32249 (18)	0.45203 (6)	0.0416 (3)	
O1	0.23316 (7)	0.20829 (17)	0.64475 (6)	0.0281 (3)	
O2	0.29956 (9)	0.1670 (2)	0.37937 (7)	0.0404 (3)	
C1	0.24718 (11)	0.2438 (2)	0.51761 (9)	0.0252 (3)	
C2	0.29950 (10)	0.4245 (2)	0.56249 (9)	0.0220 (3)	
C3	0.35600 (10)	0.5993 (2)	0.54765 (9)	0.0227 (3)	
C4	0.39264 (10)	0.7410 (2)	0.61115 (9)	0.0237 (3)	
C5	0.37560 (10)	0.7064 (2)	0.68833 (9)	0.0243 (3)	
C6	0.32286 (10)	0.5281 (3)	0.70348 (9)	0.0254 (3)	
H6	0.3123	0.5001	0.7553	0.030*	
C7	0.28666 (10)	0.3939 (2)	0.63998 (9)	0.0233 (3)	
C8	0.21110 (10)	0.1214 (2)	0.56972 (10)	0.0272 (3)	
C9	0.37842 (12)	0.6310 (3)	0.46666 (9)	0.0300 (3)	
H9A	0.3410	0.7522	0.4390	0.045*	
H9B	0.3641	0.4956	0.4355	0.045*	
H9C	0.4447	0.6657	0.4729	0.045*	
C10	0.45310 (11)	0.9320 (3)	0.59733 (10)	0.0322 (4)	
H10A	0.5185	0.8857	0.6083	0.048*	
H10B	0.4455	1.0536	0.6329	0.048*	
H10C	0.4346	0.9807	0.5418	0.048*	
C11	0.41622 (11)	0.8586 (3)	0.75636 (10)	0.0317 (4)	
H11A	0.4009	0.8035	0.8057	0.048*	
H11B	0.3900	1.0063	0.7450	0.048*	
H11C	0.4841	0.8648	0.7626	0.048*	
C12	0.15203 (12)	−0.0779 (3)	0.56170 (12)	0.0372 (4)	
H12A	0.1433	−0.1347	0.5072	0.056*	
H12B	0.0913	−0.0408	0.5733	0.056*	
H12C	0.1824	−0.1902	0.5993	0.056*	
C13	0.15085 (11)	0.4095 (3)	0.37763 (9)	0.0272 (3)	
C14	0.06721 (11)	0.4548 (3)	0.39837 (9)	0.0295 (3)	
C15	0.01129 (12)	0.6277 (3)	0.36623 (10)	0.0364 (4)	
H15	−0.0459	0.6556	0.3818	0.044*	
C16	0.04042 (13)	0.7601 (3)	0.31056 (11)	0.0398 (4)	
H16	0.0033	0.8814	0.2880	0.048*	
C17	0.12312 (13)	0.7163 (3)	0.28783 (10)	0.0390 (4)	
H17	0.1426	0.8078	0.2495	0.047*	
C18	0.17811 (12)	0.5402 (3)	0.32034 (10)	0.0338 (4)	
H18	0.2343	0.5089	0.3035	0.041*	

### Atomic displacement parameters (Å^2^)

**Table:**

	*U*^11^	*U*^22^	*U*^33^	*U*^12^	*U*^13^	*U*^23^
S1	0.0325 (2)	0.0273 (2)	0.0304 (2)	0.00289 (16)	0.00422 (17)	−0.00763 (15)
F1	0.0326 (6)	0.0485 (6)	0.0465 (6)	−0.0011 (4)	0.0151 (5)	0.0097 (5)
O1	0.0284 (6)	0.0253 (5)	0.0309 (6)	−0.0028 (4)	0.0073 (5)	0.0056 (4)
O2	0.0386 (7)	0.0465 (7)	0.0376 (7)	0.0107 (6)	0.0114 (6)	−0.0102 (6)
C1	0.0249 (8)	0.0223 (7)	0.0276 (8)	0.0023 (6)	0.0036 (6)	−0.0011 (6)
C2	0.0214 (7)	0.0218 (7)	0.0227 (7)	0.0033 (6)	0.0044 (6)	0.0013 (5)
C3	0.0209 (7)	0.0236 (7)	0.0243 (8)	0.0028 (6)	0.0063 (6)	0.0027 (5)
C4	0.0204 (7)	0.0238 (7)	0.0274 (8)	0.0018 (6)	0.0062 (6)	0.0027 (6)
C5	0.0210 (7)	0.0271 (7)	0.0241 (8)	0.0033 (6)	0.0032 (6)	0.0005 (6)
C6	0.0253 (7)	0.0305 (8)	0.0210 (7)	0.0036 (6)	0.0065 (6)	0.0045 (6)
C7	0.0202 (7)	0.0225 (7)	0.0278 (8)	0.0000 (6)	0.0065 (6)	0.0057 (6)
C8	0.0234 (8)	0.0234 (7)	0.0332 (8)	0.0010 (6)	0.0024 (6)	0.0012 (6)
C9	0.0331 (9)	0.0327 (8)	0.0258 (8)	−0.0011 (7)	0.0099 (7)	0.0024 (6)
C10	0.0307 (8)	0.0312 (8)	0.0356 (9)	−0.0071 (7)	0.0089 (7)	−0.0005 (7)
C11	0.0329 (9)	0.0338 (9)	0.0276 (8)	−0.0007 (7)	0.0042 (7)	−0.0039 (7)
C12	0.0355 (9)	0.0257 (8)	0.0497 (11)	−0.0055 (7)	0.0075 (8)	0.0027 (7)
C13	0.0264 (8)	0.0301 (8)	0.0233 (8)	−0.0002 (6)	0.0012 (6)	−0.0055 (6)
C14	0.0274 (8)	0.0339 (8)	0.0265 (8)	−0.0031 (6)	0.0042 (6)	−0.0028 (6)
C15	0.0307 (9)	0.0419 (9)	0.0354 (9)	0.0064 (7)	0.0039 (7)	−0.0050 (7)
C16	0.0438 (11)	0.0342 (9)	0.0359 (9)	0.0043 (8)	−0.0037 (8)	−0.0001 (7)
C17	0.0448 (11)	0.0409 (10)	0.0283 (9)	−0.0078 (8)	0.0008 (8)	0.0046 (7)
C18	0.0298 (9)	0.0446 (10)	0.0263 (8)	−0.0036 (7)	0.0041 (7)	−0.0024 (7)

### Geometric parameters (Å, º)

**Table:**

S1—O2	1.4839 (13)	C9—H9C	0.9800
S1—C1	1.7587 (16)	C10—H10A	0.9800
S1—C13	1.8028 (16)	C10—H10B	0.9800
F1—C14	1.3524 (19)	C10—H10C	0.9800
O1—C8	1.3660 (19)	C11—H11A	0.9800
O1—C7	1.3869 (17)	C11—H11B	0.9800
C1—C8	1.355 (2)	C11—H11C	0.9800
C1—C2	1.463 (2)	C12—H12A	0.9800
C2—C7	1.395 (2)	C12—H12B	0.9800
C2—C3	1.404 (2)	C12—H12C	0.9800
C3—C4	1.404 (2)	C13—C14	1.377 (2)
C3—C9	1.507 (2)	C13—C18	1.387 (2)
C4—C5	1.415 (2)	C14—C15	1.375 (2)
C4—C10	1.511 (2)	C15—C16	1.384 (3)
C5—C6	1.389 (2)	C15—H15	0.9500
C5—C11	1.510 (2)	C16—C17	1.378 (3)
C6—C7	1.375 (2)	C16—H16	0.9500
C6—H6	0.9500	C17—C18	1.386 (3)
C8—C12	1.479 (2)	C17—H17	0.9500
C9—H9A	0.9800	C18—H18	0.9500
C9—H9B	0.9800		
			
O2—S1—C1	111.56 (7)	C4—C10—H10B	109.5
O2—S1—C13	105.88 (8)	H10A—C10—H10B	109.5
C1—S1—C13	99.13 (7)	C4—C10—H10C	109.5
C8—O1—C7	106.45 (12)	H10A—C10—H10C	109.5
C8—C1—C2	107.46 (13)	H10B—C10—H10C	109.5
C8—C1—S1	117.25 (12)	C5—C11—H11A	109.5
C2—C1—S1	135.13 (12)	C5—C11—H11B	109.5
C7—C2—C3	118.58 (13)	H11A—C11—H11B	109.5
C7—C2—C1	103.89 (13)	C5—C11—H11C	109.5
C3—C2—C1	137.49 (14)	H11A—C11—H11C	109.5
C4—C3—C2	117.87 (13)	H11B—C11—H11C	109.5
C4—C3—C9	120.99 (13)	C8—C12—H12A	109.5
C2—C3—C9	121.13 (14)	C8—C12—H12B	109.5
C3—C4—C5	121.39 (14)	H12A—C12—H12B	109.5
C3—C4—C10	119.42 (14)	C8—C12—H12C	109.5
C5—C4—C10	119.18 (14)	H12A—C12—H12C	109.5
C6—C5—C4	120.50 (14)	H12B—C12—H12C	109.5
C6—C5—C11	118.57 (14)	C14—C13—C18	118.43 (15)
C4—C5—C11	120.91 (14)	C14—C13—S1	120.45 (13)
C7—C6—C5	116.88 (14)	C18—C13—S1	120.81 (13)
C7—C6—H6	121.6	F1—C14—C15	118.99 (15)
C5—C6—H6	121.6	F1—C14—C13	118.43 (14)
C6—C7—O1	124.37 (13)	C15—C14—C13	122.58 (16)
C6—C7—C2	124.69 (14)	C14—C15—C16	118.40 (17)
O1—C7—C2	110.94 (13)	C14—C15—H15	120.8
C1—C8—O1	111.23 (13)	C16—C15—H15	120.8
C1—C8—C12	133.80 (16)	C17—C16—C15	120.20 (17)
O1—C8—C12	114.94 (14)	C17—C16—H16	119.9
C3—C9—H9A	109.5	C15—C16—H16	119.9
C3—C9—H9B	109.5	C16—C17—C18	120.57 (17)
H9A—C9—H9B	109.5	C16—C17—H17	119.7
C3—C9—H9C	109.5	C18—C17—H17	119.7
H9A—C9—H9C	109.5	C17—C18—C13	119.79 (16)
H9B—C9—H9C	109.5	C17—C18—H18	120.1
C4—C10—H10A	109.5	C13—C18—H18	120.1
			
O2—S1—C1—C8	−134.50 (13)	C3—C2—C7—C6	2.4 (2)
C13—S1—C1—C8	114.31 (13)	C1—C2—C7—C6	−179.26 (14)
O2—S1—C1—C2	50.87 (17)	C3—C2—C7—O1	−176.94 (12)
C13—S1—C1—C2	−60.32 (17)	C1—C2—C7—O1	1.35 (16)
C8—C1—C2—C7	−1.67 (16)	C2—C1—C8—O1	1.44 (17)
S1—C1—C2—C7	173.33 (13)	S1—C1—C8—O1	−174.59 (10)
C8—C1—C2—C3	176.11 (17)	C2—C1—C8—C12	179.33 (16)
S1—C1—C2—C3	−8.9 (3)	S1—C1—C8—C12	3.3 (2)
C7—C2—C3—C4	−3.3 (2)	C7—O1—C8—C1	−0.60 (17)
C1—C2—C3—C4	179.19 (16)	C7—O1—C8—C12	−178.92 (13)
C7—C2—C3—C9	175.39 (14)	O2—S1—C13—C14	177.56 (12)
C1—C2—C3—C9	−2.1 (3)	C1—S1—C13—C14	−66.81 (14)
C2—C3—C4—C5	1.7 (2)	O2—S1—C13—C18	4.09 (15)
C9—C3—C4—C5	−176.96 (14)	C1—S1—C13—C18	119.73 (13)
C2—C3—C4—C10	−179.50 (13)	C18—C13—C14—F1	177.57 (14)
C9—C3—C4—C10	1.8 (2)	S1—C13—C14—F1	3.9 (2)
C3—C4—C5—C6	0.9 (2)	C18—C13—C14—C15	−1.9 (2)
C10—C4—C5—C6	−177.87 (14)	S1—C13—C14—C15	−175.52 (13)
C3—C4—C5—C11	179.36 (13)	F1—C14—C15—C16	−179.13 (15)
C10—C4—C5—C11	0.6 (2)	C13—C14—C15—C16	0.3 (3)
C4—C5—C6—C7	−1.8 (2)	C14—C15—C16—C17	0.7 (3)
C11—C5—C6—C7	179.67 (13)	C15—C16—C17—C18	−0.1 (3)
C5—C6—C7—O1	179.48 (13)	C16—C17—C18—C13	−1.5 (3)
C5—C6—C7—C2	0.2 (2)	C14—C13—C18—C17	2.4 (2)
C8—O1—C7—C6	−179.93 (14)	S1—C13—C18—C17	176.04 (13)
C8—O1—C7—C2	−0.54 (16)		

### Hydrogen-bond geometry (Å, º)

Cg1 and Cg2 are the centroids of the C2–C7 benzene ring and the C13–C18 2-fluorophenyl ring, respectively.

**Table:**

*D*—H···*A*	*D*—H	H···*A*	*D*···*A*	*D*—H···*A*
C6—H6···O2^i^	0.95	2.40	3.3279 (19)	165
C12—H12*C*···*Cg*1^ii^	0.98	2.69	3.486 (2)	138
C16—H16···*Cg*2^iii^	0.95	2.70	3.553 (2)	149

Symmetry codes: (i) *x*, −*y*+1/2, *z*+1/2; (ii) *x*, *y*−1, *z*; (iii) −*x*, *y*+1/2, −*z*+1/2.
